# Case Report: Novel LIM domain-binding protein 3 (LDB3) mutations associated with hypertrophic cardiomyopathy family

**DOI:** 10.3389/fped.2022.947963

**Published:** 2022-11-14

**Authors:** Junmin Zheng, Zhuangzhuang Huang, Shan Hou, Xunwei Jiang, Yongwei Zhang, Wei Liu, Jia Jia, Yun Li, Xiaomin Sun, Lijian Xie, Xiaopei Zhao, Cuilan Hou, Tingting Xiao

**Affiliations:** ^1^ Department of Cardiology, Shanghai Children’s Hospital, School of medicine, Shanghai Jiao Tong University, Shanghai, China; ^2^ Shanghai Engineering Research Center for Big Data in Pediatric Precision Medicine, Center for Biomedical Informatics, Shanghai Children’s Hospital, School of medicine, Shanghai Jiao Tong University, Shanghai, China

**Keywords:** hypertrophic cardiomyopathy, LIM domain-binding protein 3, whole exome sequencing, gene mutation, protein stability

## Abstract

Hypertrophic cardiomyopathy (HCM) is an autosomal dominant cardiomyopathy, which is one of the most common reasons for cardiac arrest in children or adolescents. It is characterized by ventricular hypertrophy (usually left ventricle), small ventricular cavity, and reduced ventricular diastolic compliance found by echocardiography in the absence of abnormal load (such as hypertension or aortic stenosis). HCM is usually caused by mutations in genes encoding sarcomere or sarcomere-related genes. Whole exome sequencing (WES) is performed to identify probable causative genes. Through WES, we identified LIM domain-binding protein 3 (LDB3) mutations (R547Q and P323S) respectively in an 11-year-old HCM girl and a 6-year-old HCM boy. Neural network analyses showed that the LDB3 (R547Q and P323S) mutation decreased its protein stability, with confidence scores of −0.9211 and −0.8967. The STRUM server also confirmed that the mutation decreased its protein stability. Thus, LDB3 mutation may be associated with heritable HCM. To our knowledge, this is the first time to report LDB3 heterozygous variants (R547Q and P323S) responsible for heritable HCM.

## Introduction

Hypertrophic cardiomyopathy (HCM) is an autosomal dominant primary myocardial disease, the characteristics of which are ventricular hypertrophy (usually left ventricle), small ventricular cavity, and reduced ventricular diastolic compliance found by echocardiography in the absence of abnormal load (such as hypertension or aortic stenosis) (1). Myocyte disorder, extracellular matrix changes, microvascular remodeling, and interstitial fibrosis are the main histological features of HCM (2, 3). The incidence rate of HCM is about 1/500–1/200, and it can affect people of any age. Many patients may have no obvious clinical manifestations such as signs or symptoms and cannot be diagnosed early. Therefore, it may be an important cause of cardiac arrest in children or adolescents (1, 4). Of course, one of the primary approaches to diagnosing HCM is transthoracic echocardiography. In addition to ventricular wall hypertrophy, it can also be combined with a series of complex manifestations, such as mitral valve abnormality and left ventricular outflow tract obstruction (5). HCM is usually caused by mutations in genes encoding sarcomere or sarcomere-related genes. More than 1,400 disease-causing genes in HCM have been identified (6). MYBPC3 and MYH7 are generally the predominant HCM disease-causing genes, accounting for 50%–70%, followed by TNNT2, TNNI3, MYL2, MYL3, TPM1, and ACTC1 (6). In addition, genes encoding Z-disk components and Ca^2+^ homeostasis factors, such as TCAP, CSRP3, ACTN2, and JPH2, can also cause HCM (6). It is well reported that most cases of HCM are caused by single-nucleotide polymorphism in genes encoding cardiac proteins (7, 8), but the mutation-related mechanism leading to HCM is not clearly understood. However, it has been reported that genetic diversity may lead to multiple gene mutations, and these genes may jointly affect the patient phenotype (9). Molecular genetic research studies provide important insights into HCM pathogenesis and support a new perspective for the diagnosis and treatment of the disease (5).

In 2014, *LDB3* gene was first reported to be closely related to HCM (10). Fratev et al. first reported a new ZASP PDZ mutation (Gly54Ser) in HCM (10). They also demonstrated that the mutation significantly affects the interactions between ZASP PDZ domains and their binding partners (10). It is suggested that ZASP^G54S^ may be related to HCM, and *LDB3* gene should be considered in general HCM gene detection (10).

In this study, we collected HCM pedigrees with two affected and five unaffected members. Whole exome sequencing (WES) was utilized to identify possible disease-causing genes or variants. Paired end reading was aligned with the GRCh37/hg19 human reference sequence. Through comprehensive ClinVar software and GATK analyses, BAM and VCF files were produced. *LDB3* gene heterozygous mutation R547Q in an 11-year-old girl and P323S in a 6-year-old boy with HCM were identified. Related families were also employed to WES in order to confirm possible disease-causing genes or variants. Our results indicate that LDB3 mutation may be associated with HCM and should be screened in prospective clinical practice to encourage early intervention.

## Materials and methods

### Clinical manifestation and examination

**Figure 1:**
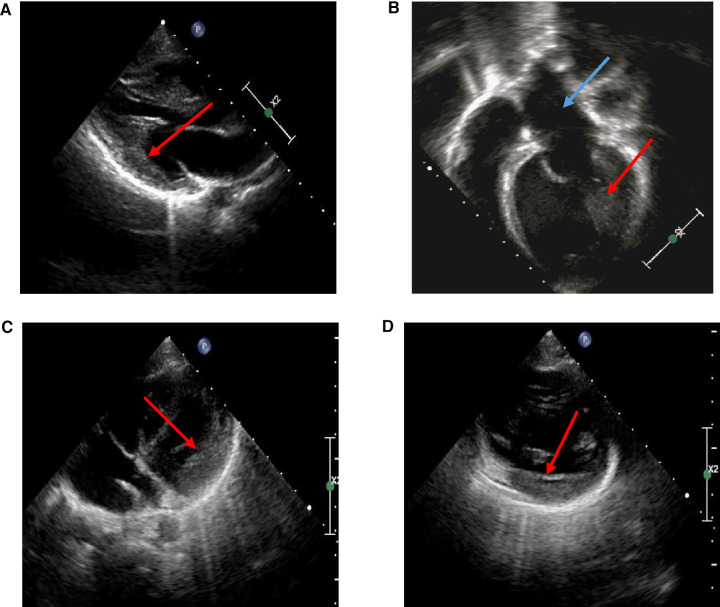
Echocardiography in HCM probands. (**A**) Echocardiography of a proband: slight left ventricular hypertrophy at later observation (red arrow). (**B**) Echocardiography of a proband: enlargement of left atrial (blue arrow) and left ventricle (red arrow) at first time. (**C,D**) Echocardiography of a proband: slight left ventricular hypertrophy (red arrow).

An 11-year-old girl was hospitalized with palpitation after exercise for 6 years. There was no special sign in her physical examination. Her 12-lead electrocardiogram (ECG) in resting time showed sinus rhythm and ventricular preexcitation, and her echocardiogram showed slight left ventricular hypertrophy after treatment (Figure 1A), although her left atrium and left ventricle were dilated, and her left ventricular ejection fraction was 25% *via* echocardiogram (Figure 1B) at the first time. The patient was given myocardial nutrition, cardiotonic diuresis, and her condition improved. Another 6-year-old boy was admitted with left ventricular hypertrophy. There was no fever, chest pain, chest tightness, palpitation, syncope, or other discomforts. There was also no special sign on his physical examination. His 12-lead ECG in resting time was normal, and echocardiogram revealed slight left ventricular hypertrophy (Figures 1C,D). He was treated with myocardial nutrition, and his condition improved. We also checked the biochemical metabolism, myocardial enzyme, and cardiac computed tomography angiography (CTA), and both were normal. The two patients should rest to avoid infection and fatigue.

### Whole exome sequencing

The construction of DNA libraries and WES were performed using the manufacturer's instructions. Genomic DNA was extracted from peripheral venous blood samples of children and their families *via* a whole-blood genomic DNA Extraction Kit (Tiangen, China). According to the manufacturer's instructions, 1 µg DNA in WES was prepared by using TruSeq DNA LT Sample Prep kit v2, hybridized with Nimblegen SeqCap EZ Exome v3 (Roche) and then matched and sequenced (2 × 100 bp) with TruSeq v3 chemistry on Illumina HiSeq 2500.

### Sanger sequencing and data analysis

LDB3 mutation was confirmed by Sanger sequencing. Brieﬂy, primers were designed that cover the mutation sequence. LDB3 mutation (R547Q) forward: 5′-CCCAACTATAACCCTGCACCCTC-3′and reverse: 5′-GCCCTGTAAACCTACTGACAAGC-3′. LDB3 mutation (P323S) forward: 5′-GCTCCCTTGACCTGTTGTCT-3′ and reverse: 5′-GGCCCTAACTACCTTGGACACA-3′. PCR products were analyzed on agarose gels and purified with the QIAqick kit (Qiagen, United States). Sanger sequencing is supported by ABI3500 (Applied Biosystems, United States) platform of Suzhou Hong Xun Biotechnology Co., Ltd.

Specifically, self-developed software was used to filter the sequencing data. Then, the filtered data were compared with a human genome database (GRCh 37/Hg 19) *via* BWA-0.710 software. Base mutations and insertion–deletion mutations in sequencing were screened and identified, compared with 370 whole exome sequencing samples in our hospital as well as 1000 Genomes Project, gnomAD, HGMD, Exome Variant Server, Exome Consortium databases, ClinVar (http:// www.ncbi.nlm.nih.gov/clinvar), and OMIM. Mutation sites were evaluated using COBALT homology alignment for evaluating amino acid sequences from different species. GERP (genomic evolutionary rate profiling), Mutation Taster (www.mutationtaster.org), combined annotation-dependent depletion (CADD), SIFT (Sorting Intolerant from Tolerant; http://siftdna.org/), and Polyphen-2 (http://genetics.bwh.harvard.edu/pph2/) were used to predict mutations among conservative species, the influence of protein structure and function. Exome Aggregation Consortium (ExAC; http://exac.broadinstitute.org) was used to test the frequency of variations. Polymerase chain reaction was used for amplification of rare mutations. Frequency between affected family members and healthy individuals and the presence or absence of variants were screened with Illumina Variant Studio. Experimental methods and data analysis were referred to the previous study (11).

### Real-time-PCR

Real-time-PCR (RT-PCR) was utilized to detect LDB3 mRNA levels. Whole-blood samples (200 µl) were used to extract RNA according to the protocol (PrimeScript™ RT Master Mix, Takara). Primers were designed to explore whether the mutation could alter its expression. Related primers are shown in Table 1.

**Table 1:** Primers used for vector construction and RT-PCR.

Primer pair	Primer sequence
LDB3-F (R547Q)	CTCTGCGAAGGTCAAGCAC
LDB3-R (R547Q)	CGGCAGGACTTGAAGCAG
LDB3-F (P323S)	CTCTGCGAAGGTCAAGCAC
LDB3-R (P323S)	CGGCAGGACTTGAAGCAG

RT-PCR, real-time-PCR.

## Results

**Figure 2:**
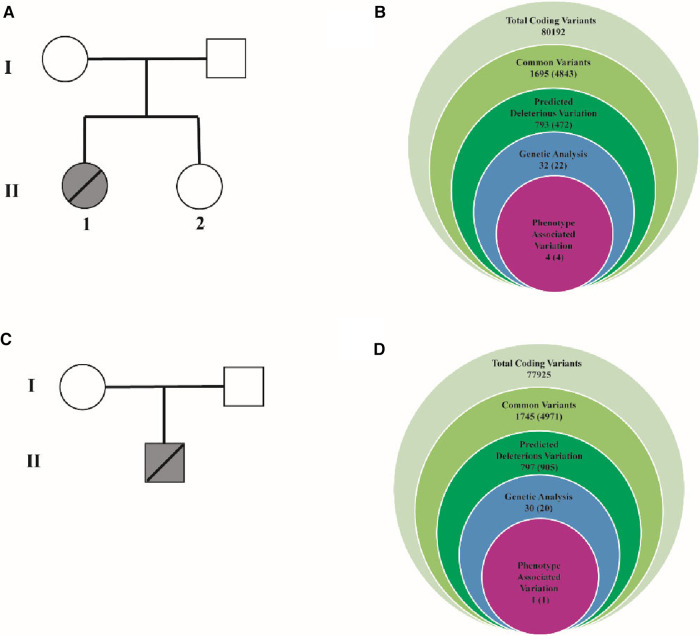
Family pedigrees and filtering processes for WES data. (**A**) One pedigree consists of one proband and three members. I-1 represents the proband's mother. I-2 represents the proband's father. II-1 represents the proband. II-2 represents the proband's sister. (**B**) The filtering process for WES data. It contains 80,192 total coding variants. Then, 1,695 common variants, 793 deleterious variations, 32 genetic analyses, and final 4 associated with this phenotype variation were filtered. TGFB3:NM_001329938:exon2:c.C487T: p.R163W, LMNA:NM_005572:exon10:c.G1712A: p.R571H, MYH7:NM_000257: exon3:c.C77T: p.A26V were deleted. (**C**) One pedigree consists of one proband. I-1 represents the proband's mother. I-2 represents the proband's father. II-1 represents the proband. (**D**) The filtering process for WES data. It contains 77,925 total coding variants. Then, 1, 745 common variants, 797 deleterious variations, 30 genetic analyses, final 1 associated with this phenotype variation were filtered.

As Figures 2A,B showed, 80,192 variants were detected and further annotated and filtered by Ingenuity Variant Analysis. According to Exome Aggregation Consortium, 1000 Genomes Project, Exome Sequencing Project, or gnomAD, 78,497 common variants were filtered and eliminated from their frequencies (minor allele frequency (MAF) < 0.01). Four variants in four genes (*TGFB3*, *LMNA*, *MYH7*, and *LDB3*) were determined and identified through a thorough analysis. Finally, LDB3 mutation was detected and selected after rigorous analysis, which was linked to the HCM phenotypes (Figure 2B). The same filtration process was undertaken with the other pedigree, and LDB3 mutation was also linked to the HCM phenotype (Figures 2C,D). These rare phenotype-related variants were classified with the American College of Medical Genetics and Genomics/Association for Molecular Pathology guidelines.

**Figure 3:**
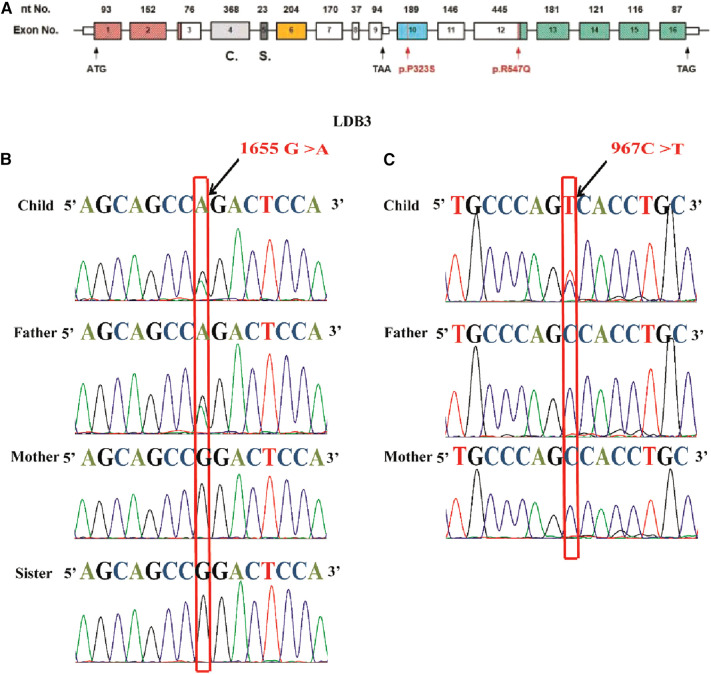
The LDB3 mutation site and Sanger sequencing. (**A**) Human *LDB3* gene maps to chromosome 10q23.2 and contains 16 exomes. The base pair mutation site is c.967C > T (p.P323S), which is located in the 10th exome of Mybcp3. The base pair mutation site is c.1655G > A, which is located in the 12th exome of Mybcp3. (**B**) The proband and her father were confirmed to be heterozygous carriers of 1655 G > A hybridization. Her mother and sister were homozygous negative of the mutation. (**C**) The other proband was confirmed to be heterozygous carriers of 967 C > T hybridization. His parents were homozygous negative of the mutation as showed through Sanger sequencing.

Heterozygous mutations of *LDB3* gene (R547Q and P323S) were identified in these two HCM pedigrees *via* WES. The mutation site c.1655G > A (p.R547Q) was located in the 12th exome of LDB3. The mutation site c.967C > T (p.P323S) was located in the 10th exome of LDB3 (Figure 3A). The proband and her father were confirmed to be heterozygous carriers of 1,655 G > A hybridization. Her mother and sister were confirmed to be homozygous negative of the mutation (Figure 3B). The other proband was confirmed to be heterozygous carrier of 967 C > T hybridization. His parents were homozygous negative of the mutation as confirmed by Sanger sequencing (Figure 3C).

**Figure 4:**
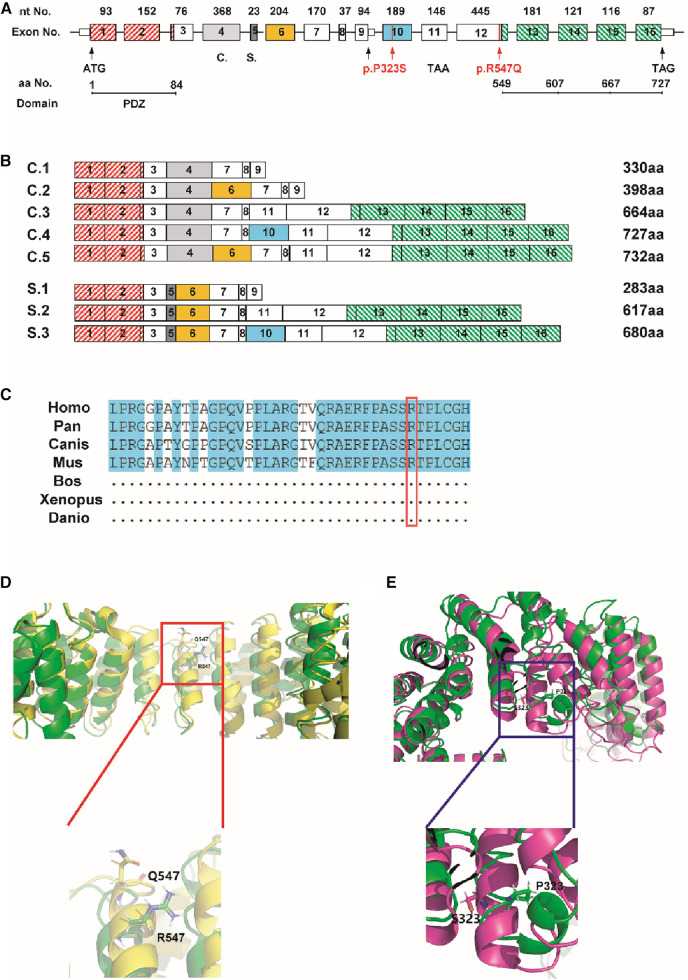
Prediction of the mutation on its functional. (**A,B**) The location of the mutation (c.967C > T, c.1655G > A) in the *LDB3* gene. (**C**) c.967C > T mutation region locates in a highly conserved area of cross-species. (**D,E**) STRUM server indicated that the P323S and R547Q mutation may affect LDB3 protein stability.

LDB3 mutants were analyzed to further explore the possibility that the function of LDB3 mutants (R547Q and P323S) affected the occurrence of diseases (Figures 4A,B). LDB3 heterozygous variant (P323S) was located in a domain of unknown function, and LDB3 heterozygous variant (R547Q) was in the LIM zinc-binding domain. Importantly, this mutation (c.1655 G > A) was located in the LDB3 conserved region (Figure 4C), indicating that the mutation may affect its protein function. Neural network analyses showed that the LDB3 (R547Q and P323S) mutation decreased its protein stability, with confidence scores of −0.9211 and −0.8967. The STRUM server also confirmed that the mutation affected LDB3 protein stability (Figures 4D,E). In addition, protein stability was confirmed by a number of web-based prediction tools such as I-Mutant and MuPRO, which also showed that the mutation affected LDB3 protein stability (Table 2). The above data indicate that LDB3 heterozygous variants (R547Q and P323S) may disrupt its protein structure and ability.

**Table 2:** Predictions using web-based tools.

Web-server	ΔΔG for mutations (kcal/mol)^a^	
	R547Q	P323S
I-Mutant3.0	−0.37	−0.45
MuPRO	−1.00	−0.70

^a^ ΔΔG < 0: destabilizing mutations; and ΔΔG > 0: stabilizing mutations.

## Discussion

In this study, we collected and analyzed the clinical phenotype and genotype of two mutated HCM pedigrees to investigate the potential disease-causing variant. Two interesting findings are as follows: (1) R547Q and P323S heterozygous mutations of LDB3 are first reported in HCM pedigrees; (2) Protein modeling assay indicates that these two LDB3 mutations (R547Q and P323S) may reduce its protein stability, which may play a crucial role for heritable HCM.

As a genetic myocardial disease, the pathogenic mechanism of HCM has not been fully understood yet. Up to now, several pathogenic hypotheses of HCM have been reported. The first theory suggests that mutations in genes encoding sarcomere proteins (such as β-MHC) and intracellular cytosomal proteins (such as titin) lead to a decrease in the rate and contraction velocity of muscle cells, alter the activity and mechanical effects of neurohumors, leading to compensatory hypertrophy of the myocardium (5). “Energy compromise” is considered a possible stimulant for the development of cardiac hypertrophy in HCM (12). Briefly, it proposed that major mutated proteins (β-MHC, cTnT, and MyBPC) cause energy depletion at certain key sites of muscle cells, a relative decrease in PCr/ATP, and are independent of cardiac hypertrophy. The third theory proposes that the mutation results in an invalid allele with haplotype dysfunction. That is, the production of normal sarcomere protein is reduced, and the composition of thick and thin sarcomere is out of balance, leading to changes in sarcomere structure and function (13, 14).

Genetic screening is an ideal tool for identification nucleotide changes that cause HCM. Although HCM has been attributed to monogenic interference in sarcomeric genes such as *MYBPC3* and *MYH7*, significant phenotypic variation is often present even in a single family (15). This variability may be attributed to gene differential expression, protein–protein interaction, and protein modification variation (15). Recently, more than 1,400 HCM-related mutations have been identified, and they are almost sarcomeric proteins and Z-disk proteins (10). Fratev et al. first reported a new ZASP PDZ mutation (Gly54Ser) in HCM, and they demonstrated that this mutation significantly affects the interactions between ZASP PDZ domains and their binding partners (10). Our results also confirmed the occurrence of *LDB3* gene mutation in HCM, and the heterozygous mutation site (R547Q and P323S) has not been reported previously. An RT-PCR assay was utilized to analyze LDB3 expression among the proband and her or his families. The LDB3 mutation site (R547Q and P323S) did not alter its mRNA expression in the two pedigrees (Tables 3, 4).

**Table 3:** Relative expression levels of LDB3 mRNA in one HCM family pedigree [LDB3 (R547Q)].

	mRNA levels
Proband (R547Q)	1
Father	1.08
Mother	1.15

HCM, hypertrophic cardiomyopathy.

**Table 4:** Relative expression levels of LDB3 mRNA in one HCM family pedigree [LDB3 (P323S)].

	mRNA levels
Proband (P323S)	1
Father	1.09
Mother	0.8

HCM, hypertrophic cardiomyopathy.

ZASP is a sarcomeric protein encoded by LDB3 and specifically expressed in human heart and skeletal muscle (16). It is well known that ZASP variants are associated with myopathies such as dilated cardiomyopathy (DCM) (17), left ventricular non-compaction cardiomyopathy (LVNC) (18, 19), and HCM (20). *LDB3* gene belongs to the PDZ-LIM domain protein family; its N-terminal contains a PDZ domain and C-terminal contains three LIM domains (21, 22). It has been reported that the PDZ domain interacts with the C terminus of α-actinin-2, and the LIM domains bind to protein kinase C and potentially have a role in protein kinase C-mediated signaling pathways (22, 23). As a sarcomeric protein, LDB3 interacts with Z-line proteins α-actinin-2, myotilin, and signaling molecules such as protein kinases (24), which indicates that LDB3 might play a crucial role in cardiomyopathy.

To further explore the impact of LDB3 variants (R547Q and P323S) on the occurrence of the HCM disease, we mapped the variant to the LDB3 protein structure. It is reported that variants locating within specific functional domains or protein translation modification sites can alter the protein function such as protein–ligand binding or protein–protein interaction (8, 25). In this study, LDB3 variants (R547Q and P323S) locate in the domain of unknown function and LIM zinc-binding domain, and the two heterozygosity mutations were both yet reported previously. It was reported that α-actinin-2 interacted with both PDZ and LIM domains (23, 26). Protein modeling assay indicates that the LDB3 mutation reduced its protein hydrophobicity and stability. Thus, the LDB3 mutation may disrupt its interaction with α-actinin-2, which plays a critical role in the heart muscle regulation. Previous research pointed that the actin-binding site in ZASP is also expressed in the heart muscle (27). Up to 20% of cardiac muscle actin consists of the skeletal muscle α-actin isoform (28). Multiple ZASP isoforms may play unique roles in maintaining the Z-disc structure by interacting with the actin in heart as well as skeletal muscle (29). The actin-binding domain is between the modular protein interacting PDZ and LIM domains. It is worth that LDB3 mutation (P323S) was located between PDZ and LIM domains. Therefore, *LDB3* gene mutation can affect the binding of this domain to actin and affect the normal contractile or diastolic function of the cardiac or skeletal muscle.

## Conclusion

Genetic pathogenic mutation and consideration of family history are necessary components of HCM diagnostic criteria. In this study, we identified two heterozygous mutation sites of LDB3 that were associated with HCM. Two pedigrees were confirmed with LDB3 heterozygous mutation (R547Q and P323S), indicating that LDB3 mutation may be a novel disease-causing variant for heritable HCM. Systematic analysis not only improves our understanding of this disease etiology but also contributes to clinical and prenatal diagnosis.

## Data Availability

The original contributions presented in the study are included in the article/Supplementary Material, further inquiries can be directed to the corresponding authors.
